# *N*-(4-Chloro­phen­yl)-4-(2-oxocyclo­pent­yl)butyramide

**DOI:** 10.1107/S1600536808042505

**Published:** 2008-12-20

**Authors:** Lin-Tao Yu, Jiang-Tao Wei, Xiang-Ge Zhou

**Affiliations:** a Institute of Homogeneous Catalysis, Department of Chemistry, Sichuan University, Chengdu 610064, People’s Republic of China

## Abstract

In the title compound, C_15_H_18_ClNO_2_, the amide group is coplanar with the chloro­phenyl group, making a dihedral angle of 1.71 (12)°. The cyclo­penta­none ring adopts a twist conformation. A weak intra­molecular C—H⋯O hydrogen bond is observed. Mol­ecules are linked into cyclic centrosymmetric dimers by paired N—H⋯O hydrogen bonds.

## Related literature

For the synthesis of cyathin terpenoids, see: Drège *et al.* (2006 ▶).
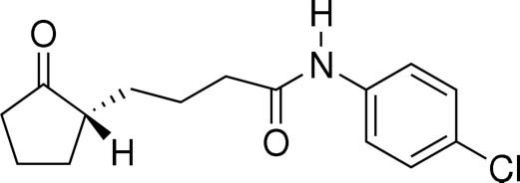


## Experimental

### 

#### Crystal data

C_15_H_18_ClNO_2_
*M* *_r_* = 279.75
Triclinic, 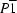

*a* = 5.5897 (2) Å
*b* = 8.8847 (3) Å
*c* = 14.6480 (4) Å
α = 80.906 (2)°
β = 86.436 (2)°
γ = 85.351 (2)°
*V* = 715.05 (4) Å^3^
*Z* = 2
Mo *K*α radiation
μ = 0.27 mm^−1^
*T* = 296 (2) K
0.42 × 0.40 × 0.20 mm

#### Data collection

Bruker SMART CCD area-detector diffractometer
Absorption correction: multi-scan (*SADABS*; Bruker, 2001 ▶) *T*_min_ = 0.895, *T*_max_ = 0.948
8899 measured reflections
3273 independent reflections
1648 reflections with *I* > 2σ(*I*)
*R*_int_ = 0.047

#### Refinement

*R*[*F* ^2^ > 2σ(*F* ^2^)] = 0.053
*wR*(*F* ^2^) = 0.147
*S* = 1.01
3273 reflections
172 parameters
H-atom parameters constrained
Δρ_max_ = 0.35 e Å^−3^
Δρ_min_ = −0.21 e Å^−3^

### 

Data collection: *SMART* (Bruker, 2001 ▶); cell refinement: *SAINT* (Bruker, 2001 ▶); data reduction: *SAINT*; program(s) used to solve structure: *SHELXS97* (Sheldrick, 2008 ▶); program(s) used to refine structure: *SHELXL97* (Sheldrick, 2008 ▶); molecular graphics: *XP* in *SHELXTL* (Sheldrick, 2008 ▶); software used to prepare material for publication: *SHELXL97*.

## Supplementary Material

Crystal structure: contains datablocks global, I. DOI: 10.1107/S1600536808042505/ci2743sup1.cif

Structure factors: contains datablocks I. DOI: 10.1107/S1600536808042505/ci2743Isup2.hkl

Additional supplementary materials:  crystallographic information; 3D view; checkCIF report

## Figures and Tables

**Table 1:** Hydrogen-bond geometry (Å, °)

*D*—H⋯*A*	*D*—H	H⋯*A*	*D*⋯*A*	*D*—H⋯*A*
N1—H1*N*⋯O1^i^	0.86	2.17	2.980 (2)	158
C15—H15⋯O2	0.93	2.29	2.889 (2)	121

Symmetry code: (i) 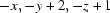.

**Fig. 1.**
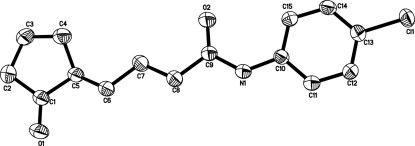
The molecular structure of the title compound, showing 30% probability displacement ellipsoids and the atomic numbering. H atoms have been omitted for clarity.

## supplementary crystallographic information

### Comment

2-Oxocyclopentyl carboxylic acid derivatives are a class of starting materials important for the preparation of cyathin terpenoids (Drège *et al.*, 2006). We report here the crystal structure of the title compound, a oxocyclopentyl derivative.

Bond lengths and angles are normal. The amide group is almost coplanar with the benzene ring system (Fig. 1). The C8/C9/N1/O2 and C10—C15 planes form dihedral angle of 1.71 (12)°. The cyclopentanone ring adopts a twist conformation. An intramolecular C15—H15···O2 hydrogen bond is observed.

The crystal packing is stabilized by N—H···O hydrogen bonds (Table 1). The molecules are linked into cyclic centrosymmetric dimers by paired N—H···O hydrogen bonds.

### Experimental

A mixture of spiro[4,4]nonane-1,6-dione (1 mmol), *p*-chloroaniline (2.2 mmol) and iodine (0.1 mmol) was stirred in refluxing dichloromethane (20 ml) for 24 h to afford the title compound. Single crystals suitable for X-ray diffraction were obtained by slow evaporation an ethyl acetate solution.

### Refinement

All H atoms were placed in calculated positions, with C-H = 0.93–0.98 Å and N-H = 0.86 Å, and refined using a riding model, with *U*_iso_(H) = 1.2*U*_eq_(C,N).

### Crystal data

**Table:**

C_15_H_18_ClNO_2_	*Z* = 2
*M**_r_* = 279.75	*F*(000) = 296
Triclinic, *P*1	*D*_x_ = 1.299 Mg m^−^^3^
Hall symbol: -P 1	Mo *K*α radiation, λ = 0.71073 Å
*a* = 5.5897 (2) Å	Cell parameters from 2436 reflections
*b* = 8.8847 (3) Å	θ = 2.8–25.4°
*c* = 14.6480 (4) Å	µ = 0.27 mm^−^^1^
α = 80.906 (2)°	*T* = 296 K
β = 86.436 (2)°	Plate, colourless
γ = 85.351 (2)°	0.42 × 0.40 × 0.20 mm
*V* = 715.05 (4) Å^3^	

### Data collection

**Table:**

Bruker SMART CCD area-detector diffractometer	3273 independent reflections
Radiation source: fine-focus sealed tube	1648 reflections with *I* > 2σ(*I*)
graphite	*R*_int_ = 0.047
φ and ω scans	θ_max_ = 27.5°, θ_min_ = 2.3°
Absorption correction: multi-scan (*SADABS*; Bruker, 2001)	*h* = −7→7
*T*_min_ = 0.895, *T*_max_ = 0.948	*k* = −11→11
8899 measured reflections	*l* = −18→18

### Refinement

**Table:**

Refinement on *F*^2^	Primary atom site location: structure-invariant direct methods
Least-squares matrix: full	Secondary atom site location: difference Fourier map
*R*[*F*^2^ > 2σ(*F*^2^)] = 0.053	Hydrogen site location: inferred from neighbouring sites
*wR*(*F*^2^) = 0.147	H-atom parameters constrained
*S* = 1.00	*w* = 1/[σ^2^(*F*_o_^2^) + (0.0664*P*)^2^] where *P* = (*F*_o_^2^ + 2*F*_c_^2^)/3
3273 reflections	(Δ/σ)_max_ = 0.001
172 parameters	Δρ_max_ = 0.35 e Å^−^^3^
0 restraints	Δρ_min_ = −0.20 e Å^−^^3^

### Special details

**Table:**

Geometry. All e.s.d.'s (except the e.s.d. in the dihedral angle between two l.s. planes) are estimated using the full covariance matrix. The cell e.s.d.'s are taken into account individually in the estimation of e.s.d.'s in distances, angles and torsion angles; correlations between e.s.d.'s in cell parameters are only used when they are defined by crystal symmetry. An approximate (isotropic) treatment of cell e.s.d.'s is used for estimating e.s.d.'s involving l.s. planes.
Refinement. Refinement of *F*^2^ against ALL reflections. The weighted *R*-factor *wR* and goodness of fit *S* are based on *F*^2^, conventional *R*-factors *R* are based on *F*, with *F* set to zero for negative *F*^2^. The threshold expression of *F*^2^ > σ(*F*^2^) is used only for calculating *R*-factors(gt) *etc*. and is not relevant to the choice of reflections for refinement. *R*-factors based on *F*^2^ are statistically about twice as large as those based on *F*, and *R*- factors based on ALL data will be even larger.

### Fractional atomic coordinates and isotropic or equivalent isotropic displacement parameters (Å^2^)

**Table:**

	*x*	*y*	*z*	*U*_iso_*/*U*_eq_	
Cl1	1.27737 (9)	0.49407 (7)	0.08887 (3)	0.06892 (18)	
O1	−0.2238 (3)	1.05919 (19)	0.73712 (11)	0.0902 (5)	
O2	0.7274 (3)	0.6356 (2)	0.50469 (10)	0.0867 (5)	
N1	0.5659 (3)	0.70953 (19)	0.36547 (10)	0.0563 (5)	
H1N	0.4405	0.7567	0.3402	0.068*	
C1	−0.0587 (4)	0.9846 (2)	0.77649 (14)	0.0629 (6)	
C2	−0.0494 (4)	0.9474 (3)	0.87916 (14)	0.0796 (8)	
H2A	−0.1708	0.8781	0.9045	0.095*	
H2B	−0.0749	1.0394	0.9075	0.095*	
C3	0.2010 (4)	0.8723 (3)	0.89563 (15)	0.0833 (8)	
H3A	0.1979	0.7899	0.9477	0.100*	
H3B	0.3091	0.9461	0.9077	0.100*	
C4	0.2785 (4)	0.8112 (3)	0.80617 (14)	0.0780 (7)	
H4A	0.4521	0.8053	0.7971	0.094*	
H4B	0.2232	0.7101	0.8075	0.094*	
C5	0.1666 (4)	0.9211 (3)	0.73132 (14)	0.0690 (7)	
H5	0.2718	1.0058	0.7195	0.083*	
C6	0.1382 (3)	0.8771 (2)	0.63830 (13)	0.0604 (6)	
H6A	0.0857	0.9677	0.5962	0.072*	
H6B	0.0131	0.8062	0.6438	0.072*	
C7	0.3644 (4)	0.8047 (3)	0.59707 (14)	0.0640 (6)	
H7A	0.4926	0.8726	0.5956	0.077*	
H7B	0.4106	0.7102	0.6369	0.077*	
C8	0.3397 (4)	0.7707 (3)	0.50060 (13)	0.0643 (6)	
H8A	0.2114	0.7029	0.5022	0.077*	
H8B	0.2928	0.8653	0.4610	0.077*	
C9	0.5627 (3)	0.6992 (2)	0.45871 (13)	0.0569 (6)	
C10	0.7424 (3)	0.6552 (2)	0.30324 (12)	0.0480 (5)	
C11	0.6974 (3)	0.6867 (2)	0.21012 (13)	0.0567 (6)	
H11	0.5551	0.7414	0.1916	0.068*	
C12	0.8597 (3)	0.6383 (2)	0.14440 (13)	0.0583 (6)	
H12	0.8272	0.6598	0.0820	0.070*	
C13	1.0715 (4)	0.5576 (2)	0.17201 (13)	0.0541 (5)	
C14	1.1172 (3)	0.5254 (2)	0.26439 (13)	0.0573 (6)	
H14	1.2602	0.4712	0.2826	0.069*	
C15	0.9538 (3)	0.5726 (2)	0.33070 (13)	0.0544 (5)	
H15	0.9854	0.5491	0.3931	0.065*	

### Atomic displacement parameters (Å^2^)

**Table:**

	*U*^11^	*U*^22^	*U*^33^	*U*^12^	*U*^13^	*U*^23^
Cl1	0.0568 (3)	0.0872 (4)	0.0638 (3)	0.0107 (3)	0.0032 (2)	−0.0254 (3)
O1	0.0787 (10)	0.1028 (12)	0.0828 (10)	0.0400 (9)	−0.0128 (8)	−0.0151 (9)
O2	0.0723 (10)	0.1230 (13)	0.0595 (9)	0.0368 (10)	−0.0164 (8)	−0.0149 (9)
N1	0.0465 (9)	0.0684 (11)	0.0526 (9)	0.0090 (8)	−0.0091 (7)	−0.0088 (8)
C1	0.0598 (12)	0.0642 (14)	0.0629 (12)	0.0108 (11)	−0.0048 (10)	−0.0112 (11)
C2	0.0792 (15)	0.0972 (18)	0.0594 (13)	0.0053 (14)	0.0041 (12)	−0.0120 (13)
C3	0.0906 (17)	0.1014 (19)	0.0553 (12)	0.0122 (15)	−0.0159 (12)	−0.0090 (13)
C4	0.0749 (15)	0.0888 (17)	0.0686 (14)	0.0144 (13)	−0.0177 (12)	−0.0118 (13)
C5	0.0677 (13)	0.0778 (15)	0.0586 (12)	0.0218 (12)	−0.0071 (11)	−0.0137 (12)
C6	0.0537 (12)	0.0688 (14)	0.0581 (11)	0.0089 (11)	−0.0066 (10)	−0.0132 (11)
C7	0.0566 (12)	0.0726 (15)	0.0634 (12)	0.0057 (11)	−0.0043 (10)	−0.0173 (11)
C8	0.0556 (12)	0.0810 (15)	0.0556 (12)	0.0094 (11)	−0.0046 (10)	−0.0148 (11)
C9	0.0502 (11)	0.0674 (14)	0.0524 (11)	0.0056 (11)	−0.0091 (10)	−0.0096 (10)
C10	0.0442 (10)	0.0490 (12)	0.0512 (10)	0.0003 (9)	−0.0041 (9)	−0.0102 (9)
C11	0.0497 (11)	0.0652 (13)	0.0543 (11)	0.0058 (10)	−0.0097 (9)	−0.0091 (10)
C12	0.0577 (12)	0.0693 (14)	0.0482 (11)	0.0029 (11)	−0.0078 (9)	−0.0117 (10)
C13	0.0524 (11)	0.0540 (12)	0.0572 (11)	−0.0021 (10)	0.0000 (9)	−0.0144 (10)
C14	0.0513 (11)	0.0565 (13)	0.0634 (12)	0.0066 (10)	−0.0109 (10)	−0.0094 (11)
C15	0.0527 (11)	0.0596 (13)	0.0505 (11)	0.0003 (10)	−0.0084 (9)	−0.0074 (10)

### Geometric parameters (Å, °)

**Table:**

Cl1—C13	1.750 (2)	C6—C7	1.508 (3)
O1—C1	1.212 (2)	C6—H6A	0.97
O2—C9	1.222 (2)	C6—H6B	0.97
N1—C9	1.353 (2)	C7—C8	1.508 (3)
N1—C10	1.410 (2)	C7—H7A	0.97
N1—H1N	0.86	C7—H7B	0.97
C1—C2	1.491 (3)	C8—C9	1.495 (3)
C1—C5	1.497 (3)	C8—H8A	0.97
C2—C3	1.517 (3)	C8—H8B	0.97
C2—H2A	0.97	C10—C11	1.383 (2)
C2—H2B	0.97	C10—C15	1.386 (2)
C3—C4	1.522 (3)	C11—C12	1.376 (3)
C3—H3A	0.97	C11—H11	0.93
C3—H3B	0.97	C12—C13	1.382 (3)
C4—C5	1.481 (3)	C12—H12	0.93
C4—H4A	0.97	C13—C14	1.374 (3)
C4—H4B	0.97	C14—C15	1.382 (3)
C5—C6	1.496 (3)	C14—H14	0.93
C5—H5	0.98	C15—H15	0.93
			
C9—N1—C10	130.17 (15)	H6A—C6—H6B	107.7
C9—N1—H1N	114.9	C8—C7—C6	113.75 (16)
C10—N1—H1N	114.9	C8—C7—H7A	108.8
O1—C1—C2	123.9 (2)	C6—C7—H7A	108.8
O1—C1—C5	126.11 (19)	C8—C7—H7B	108.8
C2—C1—C5	110.00 (17)	C6—C7—H7B	108.8
C1—C2—C3	104.75 (17)	H7A—C7—H7B	107.7
C1—C2—H2A	110.8	C9—C8—C7	114.38 (16)
C3—C2—H2A	110.8	C9—C8—H8A	108.7
C1—C2—H2B	110.8	C7—C8—H8A	108.7
C3—C2—H2B	110.8	C9—C8—H8B	108.7
H2A—C2—H2B	108.9	C7—C8—H8B	108.7
C2—C3—C4	104.48 (17)	H8A—C8—H8B	107.6
C2—C3—H3A	110.9	O2—C9—N1	122.76 (18)
C4—C3—H3A	110.9	O2—C9—C8	122.95 (18)
C2—C3—H3B	110.9	N1—C9—C8	114.29 (16)
C4—C3—H3B	110.9	C11—C10—C15	119.36 (18)
H3A—C3—H3B	108.9	C11—C10—N1	117.04 (16)
C5—C4—C3	105.72 (18)	C15—C10—N1	123.60 (16)
C5—C4—H4A	110.6	C12—C11—C10	121.07 (18)
C3—C4—H4A	110.6	C12—C11—H11	119.5
C5—C4—H4B	110.6	C10—C11—H11	119.5
C3—C4—H4B	110.6	C11—C12—C13	119.37 (18)
H4A—C4—H4B	108.7	C11—C12—H12	120.3
C4—C5—C6	120.96 (19)	C13—C12—H12	120.3
C4—C5—C1	104.34 (17)	C14—C13—C12	119.93 (18)
C6—C5—C1	115.15 (17)	C14—C13—Cl1	120.45 (15)
C4—C5—H5	105.0	C12—C13—Cl1	119.61 (15)
C6—C5—H5	105.0	C13—C14—C15	120.91 (18)
C1—C5—H5	105.0	C13—C14—H14	119.5
C5—C6—C7	113.96 (16)	C15—C14—H14	119.5
C5—C6—H6A	108.8	C14—C15—C10	119.36 (17)
C7—C6—H6A	108.8	C14—C15—H15	120.3
C5—C6—H6B	108.8	C10—C15—H15	120.3
C7—C6—H6B	108.8		
			
O1—C1—C2—C3	−172.7 (2)	C10—N1—C9—C8	−179.45 (18)
C5—C1—C2—C3	5.5 (3)	C7—C8—C9—O2	19.1 (3)
C1—C2—C3—C4	−23.3 (3)	C7—C8—C9—N1	−161.51 (18)
C2—C3—C4—C5	33.3 (3)	C9—N1—C10—C11	−178.43 (19)
C3—C4—C5—C6	−161.1 (2)	C9—N1—C10—C15	2.0 (3)
C3—C4—C5—C1	−29.4 (2)	C15—C10—C11—C12	−0.5 (3)
O1—C1—C5—C4	−166.9 (2)	N1—C10—C11—C12	179.91 (18)
C2—C1—C5—C4	14.9 (3)	C10—C11—C12—C13	−0.3 (3)
O1—C1—C5—C6	−32.0 (3)	C11—C12—C13—C14	0.5 (3)
C2—C1—C5—C6	149.9 (2)	C11—C12—C13—Cl1	179.36 (16)
C4—C5—C6—C7	−49.6 (3)	C12—C13—C14—C15	0.1 (3)
C1—C5—C6—C7	−176.49 (18)	Cl1—C13—C14—C15	−178.77 (15)
C5—C6—C7—C8	−175.97 (19)	C13—C14—C15—C10	−0.9 (3)
C6—C7—C8—C9	179.88 (18)	C11—C10—C15—C14	1.0 (3)
C10—N1—C9—O2	0.0 (3)	N1—C10—C15—C14	−179.37 (18)

### Hydrogen-bond geometry (Å, °)

**Table:**

*D*—H···*A*	*D*—H	H···*A*	*D*···*A*	*D*—H···*A*
N1—H1N···O1^i^	0.86	2.17	2.980 (2)	158
C15—H15···O2	0.93	2.29	2.889 (2)	121

Symmetry codes: (i) −*x*, −*y*+2, −*z*+1.
